# Enhancing hydatid cysts diagnosis utilizing cell-free DNA as a sensitive biomarker for *Echinococcus* spp.

**DOI:** 10.2478/helm-2026-0007

**Published:** 2026-06-15

**Authors:** S. F. SHABAN, S. A. AL-AZIZZ, M. F. ABDULHAMEED, F. H. AHMED

**Affiliations:** 1 Veterinary Hospital of Basrah, Veterinary Directorate, Ministry of Agriculture, Basrah, Iraq; 2 Department of Parasitology, College of Veterinary Medicine, University of Basrah, Basrah, Iraq; 3 *Department of Public Health, College of Veterinary Medicine, University of Basrah, Basrah, Iraq; 4 Department of Hospital Acquired Infection Control, Kirkuk Health Directorate, Kirkuk, Iraq

**Keywords:** *E. granulosus*, cfDNA, Casoni test, cysts viability, nd1 gene

## Abstract

Echinococcosis is an important zoonosis that leads to significant health problems and economic losses in livestock. The study aimed to detect cell-free DNA (cfDNA) from hydatid cysts in blood-derived samples and to determine the species/strains of *Echinococcus*. Hydatid cysts were initially collected from slaughtered sheep and tested for their viability using an eosin stain. Twenty-five rats were injected with 3000 protoscolices into the peritoneal cavity, and after a two-month observation period, they were autopsied. The Casoni test was performed to assess the hypersensitivity reaction to hydatid antigens. Polymerase chain reaction (PCR) was employed to identify *Echinococcus* spp. from multiple samples, including the cysts, sera, and plasma. The NADH dehydrogenase genes (nd1) of *E. granulosus* were targeted at 657 bp, and partial mitochondrial DNA sequences were obtained. The majority of the cysts (80%) were considered actively fertilized/viable. According to the molecular test, all studied samples were related to *E. granulosus*. The study concluded that *E. granulosus* is a common species in sheep populations in Basrah. Using cfDNA in plasma is a more efficient method for detecting hydatids than serum and faecal samples. This method is considered highly accurate and feasible for evaluating hydatid cysts in the intermediate hosts, which is critical for the control and therapy of parasitic infections.

## Introduction

Hydatid disease is a zoonotic parasitic disease caused by the larval stage of *Echinococcus*, significantly affecting humans and livestock. *Echinococcus* belongs to the family Taeniidae, immersed taxonomically within the order of Cyclophyllidea and comprises several distinct species and genotypes, typically including *Echinococcus granulosus* sensu lato (G1-G3), *E*. *equinus* (G4), *E. ortleppi* (G5), and *E. canadensis* (G6-G10) (Nakao *et al*., 2013; Maksimov *et al*., 2020). Dogs and other canids serve as definitive hosts for adult worms, while ruminants serve as intermediate hosts. Following ingestion of *Echinococcus* eggs, oncospheres are released in the intestine, penetrate the intestinal wall, and are transported through the bloodstream to various organs, primarily the liver and lungs (Badwaik *et al.,* 2024). The liver and lungs are common sites of hydatid cysts, which develop through cystogenesis. Adult animals are commonly found to harbor hydatid cysts, with the liver three times more likely to acquire the cysts than the lung or other organs (Brik *et al*., 2018; Endale & Mathewos, 2024). Extrahepatic and extrapulmonary localizations are relatively rare, with about 10 % of cases reported in the kidney, heart, brain, and eyes (Mehta *et al*., 2016; Hammami *et al*., 2019). Humans are accidentally infected with hydatids through close contact with dogs or by consuming food and water contaminated with *Echinococcus* ova. Treatment of hydatid cysts in an individual is often expensive and may require surgical intervention or long-term therapy, depending on the cyst’s anatomical site (Velasco-Tirado *et al*., 2018). Drug administration using nanotechnology, such as a combination of albendazole (polymeric nanoparticles), has been found to improve drug bioavailability and is uniquely effective as a protoscolicidal agent. According to Soleymani *et al*. (2024), treating hydatid cysts with albendazole and mebendazole nanocapsules was extremely successful.

Several diagnostic approaches are used to detect hydatid cysts in animals and humans, including imaging, serology, and molecular techniques. Serological tests such as indirect hemagglutination assays (IHA), enzyme-linked immunosorbent assays (ELISA), immunofluorescence assays (IFA), and Western blot (WB) tests have been used to identify asymptomatic cyst infections (Zait *et al*., 2020; Erganis *et al*., 2024). ELISA is usually employed as a screening test for hydatidosis in epidemiological and clinical studies, with a predictive cut-off value estimated at 30 % – 50 ^¢^%, depending on host species and pathogen strain (Chaya and Parija, 2013; Jin *et al*., 2013; Toaleb *et al*., 2023). Immunological diagnostic methods may be insufficient to distinguish between species or their related parasite strains and require highly accurate techniques, such as molecular methods, that are highly sensitive and specific (Schurer *et al.,* 2019). Many studies have suggested targeting mitochondrial genes, including cytochrome c oxidase subunit 1 (cox1) and NADH dehydrogenase subunit 1 (nd1), which are considered reliable biomarkers for identifying *Echinococcus* species and related taxa (Abulizi *etal*., 2018; Moradi *et al.,* 2019). In endemic areas, the incidence rate of human echinococcosis reached 2.3 per 100,000 inhabitants, with annual financial losses from this disease in humans and livestock estimated at USD 141,605,760 and USD 760 million, respectively (Budke *et al*., 2006; Noguera *et al.,* 2022). A lack of deworming and frequent feeding of domestic dogs infected viscera are significant risk factors for perpetuating the parasite transmission cycle in intermediate hosts. Free-roaming dogs also contribute to the spread of echinococcosis by foraging for food remnants, discarded offal, or dead carcasses, which inevitably lead to the parasite’s domestic life cycle in definitive hosts (Shamsaddini *et al*., 2024). Hydatidosis has been reported frequently across regions in Africa, the Mediterranean, South America, and Central Asia, where pastoralism is considered an integral part of the cultural heritage (Grosso *et al.,* 2012; Hogea *et al*., 2024). In the Middle Eastern societies, the incidence of hydatid cyst disease increases after Ramadan and Eid celebrations (Alishani *et al*., 2017). This trend is associated with sacrificing livestock for religious purposes and illegal slaughtering outside abattoirs, with inadequate awareness and mishandling of infected offal (Kumsa, 2019; Bekele *et al*., 2024). Free-roaming dogs may scavenge on discarded or leftover offal and thereby sustain the parasite’s life cycle (Kakamad *et al*., 2024). In Iraq, echinococcosis is prevalent, particularly in the northern and southern regions, with incidence rates of 6.3 and 4.5 per 100,000 inhabitants, respectively (Saeed *et al*., 2000; Abdulhameed *et al*., 2018). Efforts to control echinococcosis in Iraq have been inconsistent, and neither national surveys nor surveillance programs have yet been conducted (Hama & Shareef, 2016; Abdulhameed *et al*., 2019).

In epidemiological studies and the surveillance of parasitic infections, the selection of appropriate diagnostic methods is a critical option, as these tools must be both relevant and accurate to determine the presence of parasites in definitive or intermediate hosts. High diagnostic reliability, specificity, cost-effectiveness, and feasibility for use in large populations are preliminary requirements for precise estimation of disease prevalence and for making decisions about control and therapy (Borhani *et al*., 2024). Although serological tests such as ELISA and Western blot are broadly used and readily available, cross-reactivity may occur due to interactions with other parasitic infections. In addition, ELISA may not be able to distinguish among Echinococcus species in infected hosts; therefore, immunological tests alone may be insufficient for accurate diagnosis (Carmena *et al*., 2007; Hajjafari *et al.,* 2024). Nanobiosensor-based diagnostic tools have been reported to offer rapid and potentially high diagnostic accuracy. Still, a few limitations are associated with the potential toxicity, non-specific protein binding, high cost, and lack of standardization, which may retard their clinical applications to diagnose human cases (Sadr *et al*., 2023; Soleymani *et al*., 2024; Sadr *et al*., 2025). However, molecular diagnostic methods remain the gold standard due to their high specificity, ability to generate accurate results, and capacity to identify parasite species. Among these, cell-free DNA (cfDNA) analysis has emerged as a promising molecular approach for detecting parasitic infections in animals (Weerakoon & McManus, 2016; Luo *et al.,* 2024)—parasite-derived cfDNA, particularly mitochondrial DNA-targeting genes, is a non-invasive, highly species-specific method for detecting infection. cfDNA-based methods are markedly valuable for confirming active infections and conducting molecular epidemiological investigations, including species and genotype identification. Therefore, this study aimed to use molecular tools to detect mitochondrial cfDNA in hydatid cysts from patients’ blood samples and to determine the species/strains of *Echinococcus* spp.

## Materials and Methods

### Sample collection and cystsviability test

Hydatid cysts were collected from infected sheep during regular weekly visits to the slaughterhouse, where carcasses were inspected. Approximately 130 animals were found to be infected with hydatid disease. Between 50 and 60 cysts were isolated from the liver and lungs. The cysts were kept in an ice box and transported to the Parasitology Laboratory at the College of Veterinary Medicine. The outer layer of each cyst was sterilized with 70 % alcohol and carefully incised, after which the cyst fluid was decanted into Petri dishes. The cysts were examined for fertility based on the presence of protoscolices and their viability. Briefly, 1 ml of cyst fluid was mixed with 1 ml of eosin (0.1 ^¢^/o) and left for 1 – 2 minutes (Abdel-Baki *et al*., 2016). Under a light microscope, protoscolices were classified as viable if they did not take up the stain or were impervious to it, appeared green, and exhibited peristaltic movement (Aboelsoued *et al.,* 2024). In contrast, the dead protoscolices absorbed the stain and appeared red. The cyst fluid and germinal layers were preserved in sterilized tubes containing 70 % ethanol and subsequently subjected to the polymerase chain reaction (PCR) method for identification of *Echinococcus* species.

### Experimental model of hydatid infection in Rattus norvegicus

Thirty *Rattus norvegicus* (1 – 2 months old and average weight 300 grams) were used in this study. The experimental group consisted of twenty-five animals that were injected intraperitoneally with 3 000 viable protoscolices, as described by Sadeghi *et al*. (2020). The remaining five animals served as the control group. The rats were housed in steel cages and monitored daily throughout the two-month study period. After one month, the Casoni test was conducted to preliminarily assess immune sensitization to hydatid antigens, a useful indicator of successful infection (Gonlugur *et al*., 2005). Five rats were randomly selected and injected subcutaneously with 0.1 ml of fluid containing approximately 3 000 protoscolices. After one hour, the reactions were recorded; the appearance of an erythematous spot was considered a positive result.

### Detection of the development of hydatid growth

After two months, the infected rats were anesthetised with chloroform and a post-mortem examination was performed to obtain necropsy findings. This period is considered sufficient for cysts to occupy and develop within animal tissues (Ahmadi *et al*., 2020). Approximately 5 ml of blood samples were collected by cardiac puncture; half of the amount was placed into test tubes containing EDTA, and the remaining amount was poured into gel tubes. The purpose is to identify free cfDNA in plasma and serum samples from hydatids. The germinal layers were collected by carefully removing the cyst during necropsy, aspirating the cyst fluid, and gently separating the germinal layer with sterile forceps. The samples were preserved in 70 % ethyl alcohol. Also, fresh faecal pellets from rats were collected using sterile forceps and transferred into labelled containers containing 70 % ethyl alcohol.

### Genomic isolation and PCR technique for identifying Echinococcus

The genomic DNA of *Echinococcus* spp. was extracted from the germinal layer and protoscolices of the cysts, blood, and faeces (rats’ samples) using the DNeasy Blood & Tissue Kits (Qiagen, Germany). The DNA samples were stored at -20°C until the next step. The polymerase chain reaction assay (PCR) using three types of primers to amplify a partial mitochondrial locus, including NADH dehydrogenase subunit 1 (ndl) for detection of *Echinococcus granulosus*, NADH dehydrogenase subunit 1(nd1) for detection of *Echinococcus multilocularis*, and NADH dehydrogenase subunit 2 (nd2) for detection of *Echinococcus equinus*. Table 1 outlines the details regarding the targeted genes, primers, and DNA fragment sizes.

**Table 1.** Target gene and primer sets used for the detection of *Echinococcus* spp.

*Echinococcus* spp.	Target gene	Primer	5’-3’	PCR Product	Reference
*Echinococcus granulosus*	nd1	ND1-F	TGTTGCAGAGGTTTGCTGAT	674 bp	UGene lab
		ND1-R	ACGAACACGTGGTAATGTCG		
*Echinococcus multilocularis*	nd1	EMND1-1F	TTGTTCTTTGTGTTACTGTAGG	457 bp	Shang *et al*. (2019)
		EMND1-1R	CTATACAGACATTGATTACCATAA		
*Echinococcus equinus*	nd2	EEQND2-2F	CGTTAATTCACTGATACATTGTATCC	551 bp	UGene lab
		EEQND2-1R	CTCACACCAAGCACCTACAC		

The PCR operation programme started by the initial denaturation set at 94°C, 5 min, followed by 37 cycles each consisting of denaturation at 94°C, 30 seconds; annealing at 56°C, 30 sec; and extension at 72°C, 1 min; followed by a final extension at 72°C, 5 minutes. Agarose gel electrophoresis (1 ^¢^%) was used to identify amplified DNA products, which were then visualized under ultraviolet (UV) light. The successful amplified products were isolated and sent for sequencing (only four products included one blood and faecal isolate and two protoscolice isolates) to the UGene Medical Lab in Hilla, Iraq. However, using the National Centre for Biotechnology Information (NCBI) and the BLAST programme, reference sequences were obtained from GenBank. Genetic relatedness among *E. granulosus* genotypes was determined using MEGA11. The software’s programme was utilised for multiple sequence alignments; trimming was carried out, and phylogenetic analysis was performed using the phylogeny programme within the same software. Afterward, the Neighbour-Joining approach was employed to construct the phylogenetic tree from evolutionary distance data (Saitou & Nei, 1987).

## Ethical Approval and/or Informed Consent

Ethical approval was obtained from the scientific committee of the Department of Parasitology, College of Veterinary Medicine, University of Basrah (UB.VET. 23) on November 12, 2024. The experimental method was conducted according to the protocol guidelines documented by the WHO/OIE on echinococcosis in humans and animals (Eckert *et al*., 2001).

## Results

### Protoscolices and cysts examination

**Fig. 1.**
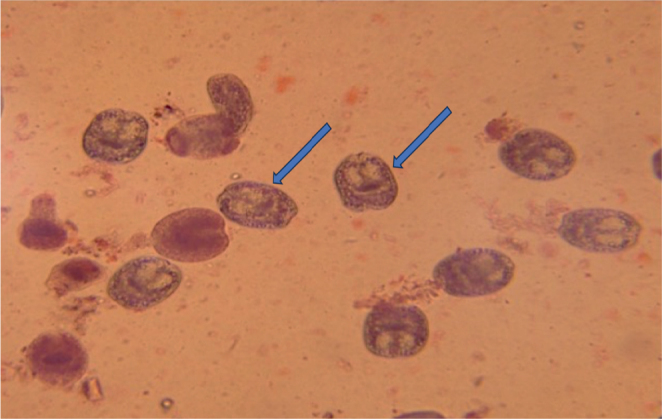
Viability of protoscolices using eosin stain (0.1 %), where stained is dead and unstained is live (blue arrow).

**Fig. 2.**
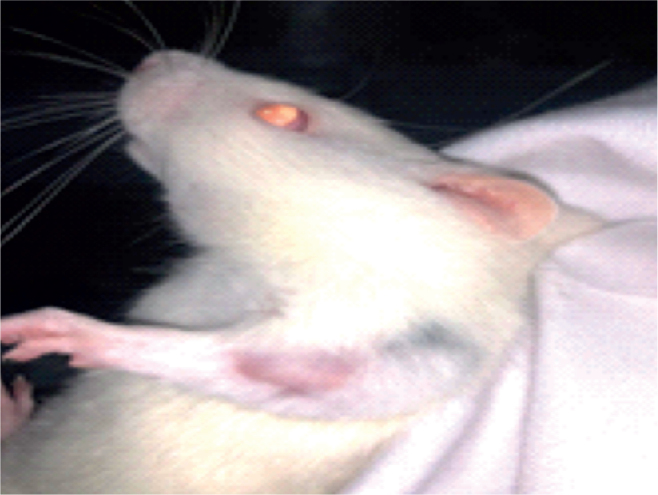
Casoni test with a hypersensitivity reaction (erythematous spot) on the upper left hand.

**Fig. 3.**
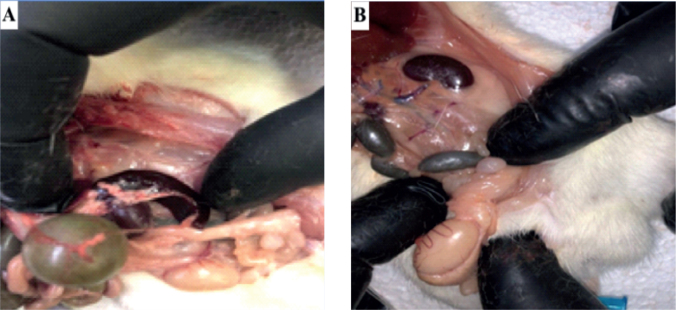
Cyst of hyadits in the viscera of the autopsied rats (A & B images).

Eighty percent (80 ^¢^%) of the protoscolices were found viable (alive) based on the stain intake and amoeboid movement (Fig. 1). According to the Casoni test, an apparent areola, or erythematous spot, was observed on the upper arm of laboratory rats (Fig. 2). Hypersensitivity reaction measuring 0.5 – 1 mm was considered positive, while a reaction area smaller than 0.5 mm was considered negative. The post-mortem examination of autopsied rats revealed small, rounded cysts, which were noticed to be developing in many organs, including the liver, lungs, and spleen (Fig. 3).

### Molecular identification of circulating cfDNA belongs to Echinococcus spp.

**Fig. 4.**
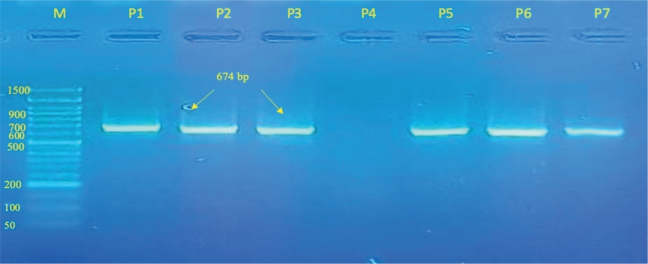
Gel electrophoresis showing the amplicon of the nd1 gene of *Echinococcus granulosus* from protoscolices samples. The DNA marker (M) is a 50-1500 bp ladder, and the six samples show a distinct 674 bp band.

**Fig. 5.**
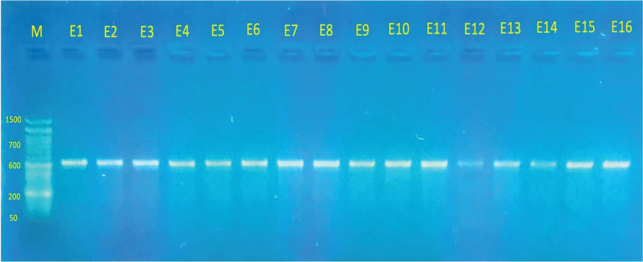
Gel electrophoresis showing the amplicon band of the nd1 gene of *E. granulosus* from the plasma samples; The DNA marker is a 50 to 1500 bp ladder, and the band size of sixteen samples is displayed as a distinct band at 674 bp.

**Fig. 6.**
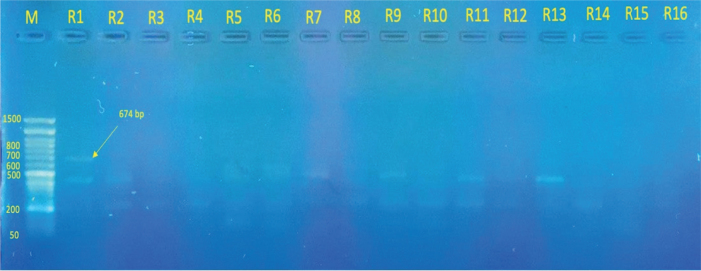
Gel electrophoresis showing the amplicon of the nd1 of *E. granulosus* from serum samples. The DNA marker (M) is a 50-1500 bp ladder, and only one sample (R1) displayed a distinct band at 674 bp. Other uncleared bands with lengths less than 500 bp may belong to DNA random fragments of *Echinococcus* in the bloodstream that are not specifically targeted.

The current study included designing specific primers to detect hydatid cysts of *E. granulosus* (674 bp, nd1 gene), *E. equines* (551 bp, nd2 gene), and *E. multilocularis* (457 bp). Out of seven samples derived from protoscolices, six were detected as *E. granulosus* by PCR (Fig. 4). Also, sixteen of the extracted plasmas were detected as *E. granulosus* by PCR amplification of the nd1 gene (Fig. 5). Of the 16 serum samples, only one successfully amplified *E. granulosus*, with an amplified band of 674 bp (Fig. 6). The fecal samples from rats were negative for all *Echinococcus* species by PCR. However, neither *E. equines* nor *E. multilocularis* was detected in the gel electrophoresis or PCR amplification products.

### Sequencing and taxa evolutionary-relationship

**Fig. 7.**
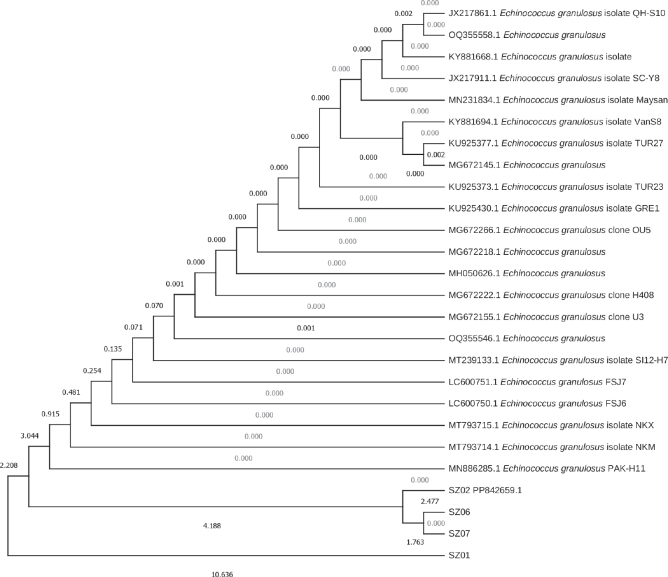
Evolutionary relationships of partial ND1 gene sequences from four isolates (sequenced; only one (SZ02) is recorded in the Gene Bank) belong to *E. granulosus.*

Only four isolates were subjected to partial mitochondrial DNA (mtDNA) sequencing. Genotyping of *E. granulosus* was deposited in GenBank under accession number SZ02 (PP842659). The branches are displayed adjacent to the ideal tree (Fig. 7). The evolutionary distances are measured in base substitutions per site and calculated using the Maximum Composite Likelihood approach (Tamura *et al*., 2004). In this analysis, 26 nucleotide sequences were examined, including codon locations in the first, second, and third positions, as well as noncoding regions. Each pair with all uncertain locations was eliminated (using the pairwise deletion option).

## Discussion

Echinococcosis is a neglected parasitic disease with public health implications and requires early diagnosis to prevent further complications (Acosta-Jamett *et al*., 2022; Paduraru *et al*., 2023). Although ultrasonography (US), computed tomography (CT), and magnetic resonance imaging (MRI) are widely used in medical and veterinary practice, they still have constraints in the differentiation of hydatid cysts from other solid lesions, including hepatic tumors, cystic neoplasms, and liver abscesses (Abbasi *et al.,* 2021; Alshoabi *et al*., 2023). In addition, calcified and the water-lily-sign cysts may be overlooked by imaging techniques, with diagnostic specificity and sensitivity declining to 70 % and 80 ^¢^%, respectively (Inan *et al*., 2007; Hołody-Zaręba *et al*., 2013). Serological assays such as ELISA and IHAT (indirect hemagglutination antibody test) are ideal screening tools in endemic areas, but their diagnostic performance remains suboptimal due to variable sensitivity and relatively low specificity, resulting from cross-reactivity with non-specific antigens (Pagnozzi *et al*., 2016; Tamarozzi *et al*., 2017; Maleki *et al.,* 2023). Lastly, nanoparticle-based biosensor platforms, including gold, silver, carbon-based, and magnetic nanoparticles, are advanced diagnostic tools having high sensitivity and rapid detection compared to classical methods. Some obstacles may limit their use in clinical applications, including the lack of standardization, nonspecific binding, physical instability, potential toxicity, and high cost (Sadr *et al*., 2025). However, molecular methods are still the gold standard for the diagnosis of parasitic infections and for species-level identification. The specificity of the cfDNA technique for detecting hydatid cyst infection has been reported to reach 100 ^¢^%, providing accurate true results (Luo *et al*., 2024; Zhao *et al.,* 2024). Our present study explores the utility of cfDNA isolated from blood samples for detecting fresh hydatid cyst infection. Unlike serological methods and biosensor platforms, which rely on host immune interaction, cfDNA analysis detects parasite-derived DNA fragments circulating in plasma or serum. This provides direct molecular evidence of active or an initial infection, eliminating false positives caused by cross-reactivity with other parasitic antigens. The Casoni intradermal test was also used as a preliminary diagnostic method to verify hydatid infection in laboratory animals (Mao *et al.,* 2019). Although widely used in epidemiological surveys in endemic regions, the Casoni test demonstrates low diagnostic performance, with reported sensitivities and specificities ranging from 47 % to 63.8 % (Ray *et al.,* 2002; Abu-Qamar *et al.,* 2004).

Hydatid cyst samples were collected from the offal of sheep, primarily from the liver and lungs, and the overall cyst viability was estimated to be approximately 80 %. Intrinsic factors, including host species and age, immune response to protoscolices, and cyst anatomical location, primarily influence the fertility and viability of hydatid cysts. Similar findings have been reported in other endemic regions, including Libya and Iran, where cyst viability in sheep and goats was estimated at around 80 % (Elmajdoub & Rahman, 2015; Shabani *et al*., 2022). In contrast, hydatid cysts in cattle often exhibit lower viability, with many cysts are usually degenerated or calcified (Getachew *et al*., 2012). This reduced viability is largely attributed to a stronger cellular immune response that limits cyst growth in cattle and buffalo (Hidalgo *et al.,* 2019). Additionally, host-parasite adaptation may influence the development of multiple cysts and their viability in different intermediate hosts.

Using PCR, the species *E. granulosus* was identified in protoscolices from different fertile cysts isolated from slaughtered sheep. This may be explained by the fact that *E. granulosus* is closely associated with specific animal species and geographic regions, where the parasite’s domestic life cycle is maintained in dogs and sheep. In northern Iraq, *E. granulosus* was found in 32.4 % of cyst lesions in sheep (Hamoo *et al.,* 2019), while the prevalence in stray dogs was estimated at 24.25 % (Aziz *et al*., 2022). A previous study confirmed that *E. granulosus* was identified in 62 patients admitted to Duhok hospitals for cyst removal surgery; the majority (54 ^¢^%) were residents of rural areas (Issa *et al.,* 2022). *E. granulosus* has been often reported in some neighbouring countries, including Iran and Turkey, with a prevalence rate estimated of 11.34 % and 31.7 ^¢^%, respectively (Shabani *et al.,* 2022; Avcioglu *et al.,* 2022). Frequent deworming of dogs and increased health awareness about hydatids are cornerstones of the intervention programme to reduce disease incidence in endemic regions.

The serum and plasma samples were examined for *Echinococcus* cfDNA, with plasma demonstrating excellent diagnostic performance. All plasma samples tested positive in gel electrophoresis showed amplification of 674 bp bands, whereas serum samples showed lower sensitivity for diagnosis. The sensitivity of plasma for cfDNA detection was 62.5 ^¢^%, and its specificity was 100 ^¢^%, making it more feasible than serum, which exhibited sensitivity between 20.0 % and 23.8 % (Zhao *et al.,* 2021). Habibi *et al*. (2024) highlighted the promise of plasma as a biomarker for cfDNA-based diagnosis of echinococcosis, achieving a 79 % validation rate in patients with hydatid infections, making it a more accurate choice than urine samples. According to a study by Hadipour *et al*. (2023), the cfDNA sensitivity for detecting cystic echinococcosis in sheep using serum-based PCR may be increased to 95 % when a large volume of serum is obtained from infected animals. The detection of DNA fragments in echinococcosis is an ideal screening method in many endemic regions, surpassing immunodiagnostic tests in diagnostic precision and in its ability to provide insight into hydatid phenotypic traits (Wan *et al*., 2020; Fan *et al*., 2021).

To further confirm species identity, a phylogenetic analysis was constructed using partial mitochondrial DNA sequences. The analysis of the 351 bp fragment of the nd1 gene revealed close genetic affiliations with regional *E. granulosus* strains, including the isolates from Iran, Pakistan, and China, with a minimum genetic distance of 0.001. Prior studies focused on gene and haplotype characterizations have documented a potential genetic diversity network within *E. granulosus*, particularly among the G1-G3 genotypes, which were widely reported across numerous Asian and European countries (Hassan *et al*., 2017). In Iraq, the G1-G3 genotype is considered the dominant strain of *E. granulosus* and is consistently detected in sheep and humans (Al-Asadi *et al*., 2021; Issa *et al*., 2022). The isolates examined in this study showed 0.13 % similarity to additional isolates (LC600750.1) previously reported from Maysan province (Yaseen *et al*., 2022). Al-Asadi *et al*. (2024) found a common lineage among *E. granulosus* isolates from the provinces of Basrah and Maysan, characterized by 11 amino acid substitutions in the mtDNA sequence. Our study, however, demonstrates a promising approach that uses the plasma abundance of cfDNA to detect echinococcosis, thereby enabling the estimation of prevalence in livestock and humans. Nonetheless, the study acknowledges some limitations, including the need to sequence more isolates and to identify the divergent *E. granulosus* genotypes. Addressing these limitations will be incorporated into future research programmes for the diagnosis/identification of hydatid cyst infection in livestock.

## Conclusions

The present study used plasma and serum samples to detect hydatid cyst infection by identifying circulating cfDNA in a rat-based laboratory model.

Plasma samples showed higher sensitivity for detecting hydatid cysts than serum and faecal samples, indicating better diagnostic performance and more reliable results. This cfDNA-based approach shows promise for identifying hydatid cysts and their species in animals and humans. The use of cfDNA as a molecular diagnostic tool offers accurate results that can support control programs and select appropriate therapies. Partial sequencing of the mitochondrial nad1 gene was also performed, providing valuable insights into parasite-host relationships, the spread of parasite genotypes, and genetic divergence of *Echinococcus* in Basrah. The findings support expanding this diagnostic approach to livestock and herd-level surveillance to better understand the epidemiological status of hydatid disease in Basrah Province. Such data are important for establishing baseline information and strengthening control strategies within a One Health framework. However, we acknowledged that some limitations should be addressed, including the study’s lack of comparison with other conventional methods, such as serological tests, which provide figures for the sensitivity and specificity of both tests. The study assessed cfDNA from hydatids at a specific time, should have included more replicates, and evaluated at various stages of infection (early, developing, and chronic). Further studies involving naturally infected hosts are needed to validate the clinical application of this approach for the diagnosis of hydatid cysts in intermediate hosts.
